# Neural Coding in Spiking Neural Networks: A Comparative Study for Robust Neuromorphic Systems

**DOI:** 10.3389/fnins.2021.638474

**Published:** 2021-03-04

**Authors:** Wenzhe Guo, Mohammed E. Fouda, Ahmed M. Eltawil, Khaled Nabil Salama

**Affiliations:** ^1^Sensors Laboratory, Advanced Membranes and Porous Materials Center (AMPMC), Computer, Electrical, and Mathematical Sciences and Engineering (CEMSE) Division, King Abdullah University of Science and Technology, Thuwal, Saudi Arabia; ^2^Communication and Computing Systems Laboratory, Computer, Electrical, and Mathematical Sciences and Engineering (CEMSE) Division, King Abdullah University of Science and Technology, Thuwal, Saudi Arabia; ^3^Department of Electrical Engineering and Computer Science, University of California, Irvine, Irvine, CA, United States

**Keywords:** neural codes, rate coding, burst coding, phase coding, time to first spike coding, spiking neural networks, unsupervised learning, neuromorphic computing

## Abstract

Various hypotheses of information representation in brain, referred to as neural codes, have been proposed to explain the information transmission between neurons. Neural coding plays an essential role in enabling the brain-inspired spiking neural networks (SNNs) to perform different tasks. To search for the best coding scheme, we performed an extensive comparative study on the impact and performance of four important neural coding schemes, namely, rate coding, time-to-first spike (TTFS) coding, phase coding, and burst coding. The comparative study was carried out using a biological 2-layer SNN trained with an unsupervised spike-timing-dependent plasticity (STDP) algorithm. Various aspects of network performance were considered, including classification accuracy, processing latency, synaptic operations (SOPs), hardware implementation, network compression efficacy, input and synaptic noise resilience, and synaptic fault tolerance. The classification tasks on Modified National Institute of Standards and Technology (MNIST) and Fashion-MNIST datasets were applied in our study. For hardware implementation, area and power consumption were estimated for these coding schemes, and the network compression efficacy was analyzed using pruning and quantization techniques. Different types of input noise and noise variations in the datasets were considered and applied. Furthermore, the robustness of each coding scheme to the non-ideality-induced synaptic noise and fault in analog neuromorphic systems was studied and compared. Our results show that TTFS coding is the best choice in achieving the highest computational performance with very low hardware implementation overhead. TTFS coding requires 4x/7.5x lower processing latency and 3.5x/6.5x fewer SOPs than rate coding during the training/inference process. Phase coding is the most resilient scheme to input noise. Burst coding offers the highest network compression efficacy and the best overall robustness to hardware non-idealities for both training and inference processes. The study presented in this paper reveals the design space created by the choice of each coding scheme, allowing designers to frame each scheme in terms of its strength and weakness given a designs’ constraints and considerations in neuromorphic systems.

## Introduction

Artificial neural networks (ANNs) have achieved state-of-the-art results in various applications ranging from computer vision (Krizhevsky et al., 2017), speech recognition (Graves and Schmidhuber, 2005), to natural language processing (Collobert et al., 2011). However, the great success comes at the cost of massive large-scale computational operations and high energy consumption (Han et al., 2015). Spiking neural networks (SNNs) have attracted ever-growing attention from research communities for its superior energy efficiency. Inspired by the biological nervous system, SNNs transmit and process the information on the occurrence of a spike or an event generated by a neuron, the central computing unit. The large spike sparsity and simple synaptic operations (SOPs) in the network enable SNNs to outperform ANNs in terms of energy efficiency. The computing capability of SNNs has been explored in a broad range of applications, such as pattern recognition (Sengupta et al., 2019), object detection (Zhou et al., 2020), navigation (Koul and Horiuchi, 2019), and motor control (Naveros et al., 2020). Recently, various neuromorphic computing systems built on SNNs have been proposed to solve the bottleneck posed by the traditional Von Neumann computing systems (Furber et al., 2014; Davies et al., 2018; Frenkel et al., 2019). Their massive parallelism, asynchronous event-driven operations, and distributed memory provide huge potential in accelerating information processing and reducing energy consumption in many applications.

The human brain is by far the most complex, sophisticated, and energy-efficient computing system. Its remarkable computational power is realized through the interaction and communications among neurons, the primitive processing units, which transmit information between each other through trains of action potentials (spikes). It is well known that sensory information is encoded in the spike patterns. A neural code refers to the neural representations of information in a pattern. Neural coding schemes of spike patterns have been extensively studied to unveil the mystery of our cognitive systems and provide the underlying fundamentals of information transmission and processing (Gerstner et al., 1997; Li and Tsien, 2017; Azarfar et al., 2018).

Various coding methods have been proposed to explain the information encoding mechanism, such as rate coding (Adrian and Zotterman, 1926), temporal coding, phase coding, and burst coding. Rate coding utilizes spiking rates to represent information, and it has been a dominant paradigm in neuroscience and ANNs for decades because of its robustness and simple mechanism. Rate coding has been experimentally discovered in most sensory systems, such as visual cortex and motor cortex (Srivastava et al., 2017). However, rate coding scheme is limited by a lengthy processing period and slow information transmission. To explain efficient and fast response mechanism in our brain, temporal coding was hypothesized as a neural code that uses the precise spike timing to convey information in different forms, such as the timing of the first spike (Johansson and Birznieks, 2004), the rank order between spikes (Thorpe and Gautrais, 1998), and relative spike latency (Gollisch and Meister, 2008). Time to first spike (TTFS) coding scheme transmits information to the destination neurons on the arrival of the first spike, which enables a super-fast transmission speed. Many experiments have pointed out the significance of the first spikes in various parts of our nervous system, such as retina, auditory systems, and tactile afferents (Ponulak and Kasinski, 2011). Various works have reported that applying TTFS coding scheme in SNNs can significantly reduce the number of spikes and improve inference speed (Rueckauer and Liu, 2018; Oh et al., 2020; Park et al., 2020). Phase coding encodes information in spike patterns whose phases are correlated with internally generated background oscillation rhythms, which has been experimentally observed in the hippocampus and olfactory system (O’Keefe and Recce, 1993; Laurent, 1996). Faster inference speed was reported to be achieved by phase coding compared with rate coding in SNNs (Kim et al., 2018c). It has been widely observed that neurons also communicate with each other in a burst of spikes in various parts of the nervous system, such as thalamus cortex, hippocampus, and auditory system, which gives rise to the hypothesis of burst coding (Zeldenrust et al., 2018). Burst spikes were demonstrated to be more reliable in information transfer and contain more information, which can lead to high energy efficiency and fast processing speed in SNNs (Park et al., 2019).

Recent studies compared different neural coding schemes in inference performance in SNNs converted from well-trained deep neural networks (DNNs) (Park et al., 2019, 2020). The TTFS-based temporal coding was demonstrated to exhibit the highest classification accuracy, fastest inference speed, and lowest energy consumption. Its superior performance was attributed to the only one spike operation and the utilization of precise timing. However, these works used a DNN-converted SNN and only showed the comparison in inference performance at the algorithmic level. The impact and performance of different neural coding methods on the training process have yet to be investigated, which is vital for designing an online learning system. They also failed to provide a comparison from hardware implementation perspectives. For example, noise resilience, fault tolerance, and implementation overhead are essential considerations in designing a real-time neuromorphic system. Therefore, in this work, we present a comparative study of the impact and performance of different neural coding schemes from various aspects of design during both the training and inference processes. Four neural coding schemes, namely, rate coding, TTFS coding, phase coding, and burst coding, are chosen for their importance in understanding the underlying information encoding mechanism and their important roles in many parts of nervous systems. The performance is evaluated in terms of classification accuracy, processing latency, SOPs, hardware implementation, network compression efficacy, noise resilience, and fault tolerance.

The main contributions of our work are summarized as follows.

• We discuss the important neural coding schemes and propose a simple and effective burst coding scheme that applies a burst of spikes for information transmission, which is proven to be fast and robust.
• We investigate and analyze the impact of different neural coding schemes on the performance of a SNN in various aspects for training and inference processes.
• We present a comprehensive comparison among different coding schemes in terms of classification performance, computational performance, hardware implementation, network compression efficacy, noise resilience, and fault tolerance.
• The selection of the best coding scheme is discussed for achieving the best performance of neuromorphic systems under different design constraints.

## Background and Methods

### Neural Models and Network Architecture

To model spiking neurons, the leaky integrated-and-fire (LIF) model was used in this work because of its computational efficiency and capability of capturing the essential features of information processing in the nervous system (Burkitt, 2006). The model consists of one first-order linear differential equation that defines the dynamics of membrane potential and a threshold condition that determines the generation of spikes (Guo et al., 2020a). Synapses serve as the transmission medium that permits the signals (electrical or chemical) to be passed from one neuron to the target neuron. They are modeled as conductance with time-varying dynamics. Spike-timing-dependent plasticity (STDP) relates the synaptic plasticity to the relative timing difference between a presynaptic spike and postsynaptic spike. A simplified STDP was used to update synaptic weights (Masquelier and Horpe, 2007), which is described by

(1) $$\text{Δ}\,w_{i\,j}=\left\{ \begin{array}{c} \mu_{+}\,w_{i\,j}\,\left(1-w_{i\,j}\right),i\,f\,t_{j}-t_{i}<0,\\ \mu_{-}\,w_{i\,j}\,\left(1-w_{i\,j}\right),i\,f\,t_{j}-t_{i}>0. \end{array}\right.$$

where *w*_*ij*_ is the synaptic weight between a presynaptic neuron *j* and a postsynaptic neuron *i*, *t*_*j*_, and *t*_*i*_ are the firing time of the presynaptic neuron *j* and the postsynaptic neuron *i*, respectively, μ_+_ and μ_−_ are the learning rates. A learning time window was used so that the presynaptic spikes that were located outside the window with respect to a postsynaptic spike have no relation with the postsynaptic spike.

**FIGURE 1:**
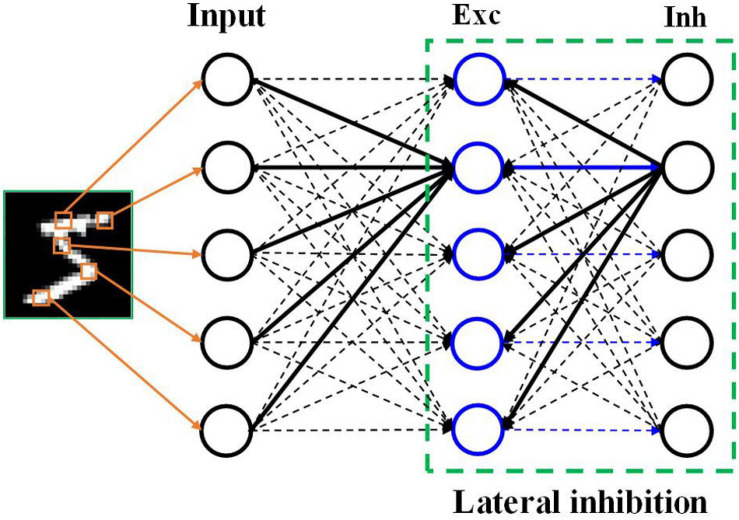
SNN architecture. The input layer encodes the input pixels into spikes and is fully connected to the excitatory (Exc) neuron layer. The processing layer follows a winner-take-all principle with a special connection pattern between excitatory neurons and inhibitory (Inh) neurons, which induces lateral inhibition effect.

A two-layer SNN architecture was adopted and tested on the Modified National Institute of Standards and Technology (MNIST) dataset and Fashion-MNIST dataset (Lecun et al., 1998; Diehl and Cook, 2015; Xiao et al., 2017). As shown in Figure 1, this architecture consists of an input layer and a processing layer. The input layer has 784 units, each of which converts an input pixel into spikes using different neural coding schemes. The input layer is fully connected to the processing layer. In the processing layer, 100 excitatory neurons were used, which send spikes to inhibitory neurons in a one-to-one fashion, whereas each inhibitory neuron sends spikes to all the excitatory neurons except the one that it receives spikes from. This connection pattern implements a winner-take-all (WTA) mechanism, which imposes lateral inhibition on excitatory neurons and hence competitions for learning input features. To ensure fair competition, a threshold adaption scheme is applied. Whenever a neuron fires, its threshold is increased by an adaption constant and then slowly decays with time. The phenomenon of threshold adaption has been commonly observed in the central nervous system. A simple classification scheme is implemented based on the firing activity of excitatory neurons. After training, excitatory neurons are assigned labels to which they fire the most spikes. They are then divided into 10 groups, each of which corresponds to a digit and contains all the neurons labeled by this digit. During inference, the classification result for an input image is the digit of the group with the highest average spike counts. All the simulations in this work were run in a Python-based platform.

### Coding Schemes

**FIGURE 2:**
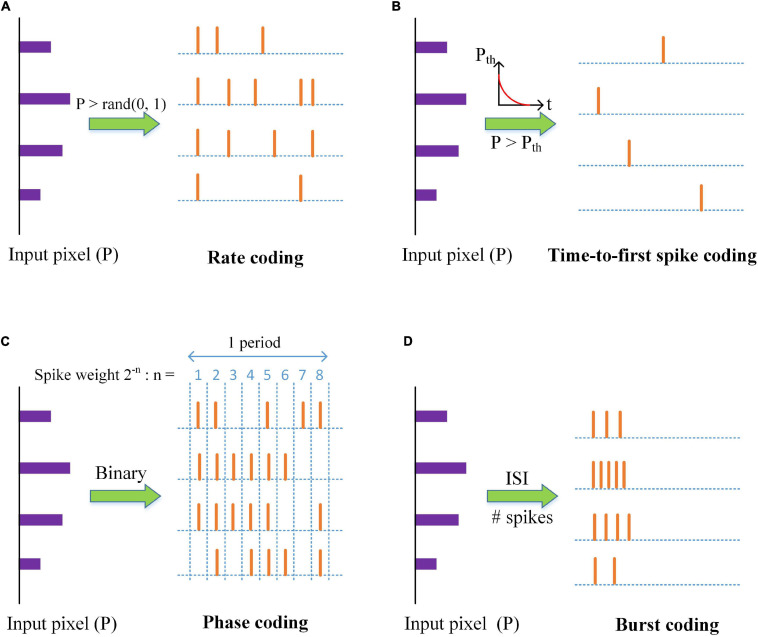
An illustration of neural coding schemes; **(A)** Rate coding, **(B)** Time-to-first spike coding, **(C)** Phase coding, and **(D)** Burst coding. P is the value of an input pixel. ISI is the inter-spike interval.

Neural coding schemes are used to convert input pixels into spikes that are transmitted to the excitatory neurons. Four different types of neural coding schemes were studied and compared, namely, rate coding, TTFS coding, phase coding, and burst coding. The operation principles of the coding schemes are illustrated in Figure 2. The details of these schemes are explained in the following subsections. It is worth mentioning that these coding schemes are used only for input data encoding and the output neurons are regular LIF neurons, not encoded.

### Rate Coding

Rate coding is the most widely used coding scheme in neural network models. This scheme considers each input pixel as a firing rate and converts the pixel into a Poisson spike train with the firing rate. Input pixels are scaled down by a factor λ. The factor is selected as four for the optimal classification and computational performance, and the corresponding firing rates are confined between 0 and 63.75 Hz. As shown in Figure 2A, Poisson spike trains were generated by comparing the scaled pixels with random numbers.

### Time-to-First-Spike Coding

Time to first spike coding was discovered to encode information for fast responses within a few milliseconds, like tactile stimulus (Johansson and Birznieks, 2004), by using the first spikes. Park et al. (2020) proposed a fast and energy-efficient TTFS coding scheme that used an exponential-decaying dynamic threshold to convert input pixels to the first-spike patterns. The larger an input pixel is, the more information it carries, and the earlier it emits a spike. The input pixels are first normalized through the division by the maximum value. An exponential function is used to compute the threshold *P*_*th*_, described by *P*_*th*_(*t*) = θ_0_exp(−*t*/τ_*th*_), where θ_*0*_ is a threshold constant and set as 1, and τ_*th*_ is the time constant. A spike is generated when the input pixel exceeds the threshold, and the input is inhibited from generating more spikes, as shown in Figure 2B. In this scheme, the input pixels are translated into the exact timing of the first spikes.

The precise times of the input spikes are used to decode the amount of information that input spikes deliver to the post-synaptic neurons in the decoding phase. The input spikes excite the synapse to produce synaptic input in form of the sum of post-synaptic potentials (PSPs) as below

(2) $$z_{j}\,\left(t\right)=\sum_{i}P\,S\,P_{i\,j}\,\left(t\right)=w_{s}\,\left(t\right)\,\sum_{i}w_{i\,j}\,s_{i}\,\left(t\right)$$

where *z*_*j*_(*t*) is the synaptic input to the postsynaptic neuron j, *PSP*_*ij*_(*t*) is the post-synaptic potentials resulting from the input neuron *i*, *s*_*i*_(*t*) is the input spike train from the presynaptic neuron *i*, *w*_*ij*_ is the synaptic weight. *w*_*s*_(*t*) is the spike weight at time *t* and it is an exponentially-decaying function [i.e., w_s_(t) = exp(−t/τ_s_)]. The PSP is in a simplified form and has been widely adopted (Kheradpisheh et al., 2018; Kim et al., 2018c; Lee et al., 2019). The input neuron contributes to the postsynaptic neuron only at its firing time and its effect is not accumulated in time. This simplified form eliminates the need for vector matrix multiplication in the network, which can significantly improve hardware system performance. The spike weighting ensures that different firing times lead to different amount of information. The earlier a spike arrives, the larger weight it carries, and the more information it transmits to the post-synaptic neurons. The decoding process is necessary to ensure the precise transmission of input information. The post-synaptic neurons accumulate their membrane potentials by integrating the synaptic input and output spikes once the potentials reach the firing threshold.

### Phase Coding

Kim et al. (2018c) proposed a simple phase coding scheme by converting input pixels into their binary representation where the bit, “1,” signals a spike, as shown in Figure 2C. The phase information is added to the spikes by assigning different weights to each bit in the representation. The number of the phases is 8, determined by the largest pixel intensity (255). The spike weight changes with time periodically, given by *w*_*s*_(*t*) = 2^−[(1 + *mod*(*t*−1, 8))]^, which represents the significance of each bit in the binary representation. The decoding phase uses the weighted spikes to produce the synaptic input, described by Equation (2). The larger an input pixel is, the more significant spikes it produces, and the more information it transmits.

### Burst Coding

Burst coding enables fast and efficient information transmission by sending a burst of spikes at one time. Sending a burst of spikes instead of a single spike can increase the reliability of synaptic communication between neurons. It has been demonstrated that in burst coding, information is carried in the number of spikes (N_*s*_) and the inter-spike interval (ISI) in the burst (Izhikevich et al., 2003; Eyherabide et al., 2009). Accordingly, we propose a simple and effective burst coding scheme that converts input pixels into spike bursts with the number of spikes and ISI proportional to the pixel intensities. The conversion is illustrated in Figure 2D. First, input pixels are normalized in the range from zero to one. For an input pixel P, the number of spikes in a burst is calculated as *N*_*s*_(*P*) = ⌈*N*_*max*_*P*⌉, where *N*_*max*_ is the maximum number of spikes and ⌈⋅⌉ is the ceiling function. The conversion of ISI is given by

(3) $$I\,S\,I\,\left(P\right)=\left\{ \begin{array}{c} \left\lceil -\left(T_{m\,a\,x}-T_{m\,i\,n}\right)\,P+T_{m\,a\,x}\right\rceil ,N_{s}>1,\\ T_{m\,a\,x},o\,t\,h\,e\,r\,w\,i\,s\,e. \end{array}\right.$$

where *T*_*max*_ and *T*_*min*_ are the maximum and minimum intervals, respectively. The ISI is confined in [*T*_*min*_,*T*_*max*_]. A larger input pixel produces a burst with a smaller ISI and more spikes inside. The parameters are configured in a biological range. *N*_*max*_ is chosen as five for the optimal classification and computational performance, and more than five spikes in a burst are very rare in a biological system (Buzsáki, 2012). *T*_*max*_ was chosen as the time window for processing one image. *T*_*min*_ was taken as 2 ms (Reich et al., 2000).

The network parameters used in our simulation are associated with LIF neuron model, synaptic model, and STDP. The network parameter settings for all the coding schemes are shown in Table 1. The model parameters associated with each coding scheme are listed in Table 2. Due to a large parameter dimension, manual tuning is not reliable and grid search is very time-consuming. Thus, we used genetic algorithm (GA) to optimize the parameters together for SNNs with each coding scheme to achieve sufficient classification accuracy. In rate coding, the scaling factor λ is selected separately for the optimal classification and computational performance, as its impact is more significant. The same optimization applies to the maximum number of spike *N*_*max*_ in burst coding. The optimization detail is discussed in section “Classification and Computational Performance.” The maximum and minimum intervals in burst coding are selected carefully so that the burst spike frequency is confined in a reasonable and biological range. In TTFS coding, the two time constants are optimized together with the network parameters by GA, as they have smaller impact on the computational performance.

**TABLE 1:** SNN parameters used in the simulation for each coding scheme.

SNN parameters	Description	Values for different coding schemes			
		Rate	TTFS	Phase	Burst
τ_*m*_,*V*_*th*_	Time constant and firing potential threshold in LIF model.	10 ms, 0.6 mV	10 ms, 0.5 mV	10 ms, 0.8 mV	10 ms, 0.4 mV
τ_*g*_	Time constant in synaptic conductance model.	30 ms	10 ms	30 ms	30 ms
μ_−_,μ_+_	Learning rates in STDP model.	0.002, 0.02	0.0004, 0.09	0.006, 0.004	0.001, 0.07
θ_+_	Firing threshold adaption constant.	0.008 mV	0.04 mV	0.008 mV	0.005 mV

**TABLE 2:** Model parameters of each coding scheme used in the simulation.

Coding scheme	Rate	TTFS	Phase	Burst
Parameters	λ	τ_*th*_,τ_*s*_	NA	*t*_*max*_,*t*_*min*_,*N*_*max*_
Values	4	6 ms, 15 ms	NA	10 ms, 2 ms, 5

## Results and Discussion

### Input Spike Patterns

**FIGURE 3:**
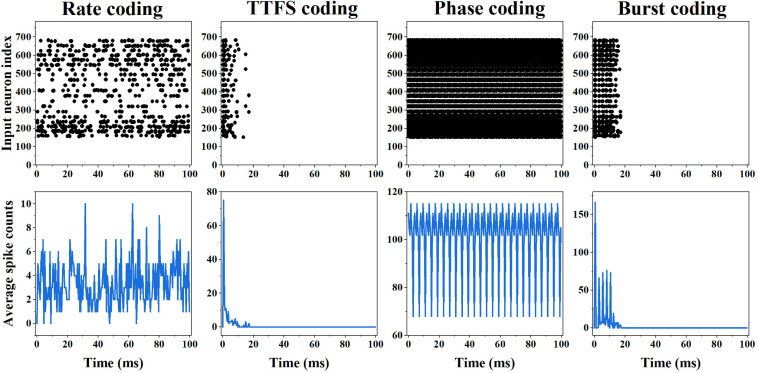
First row: input spike patterns of an example input digit five for different coding methods in 100 ms time window. Second row: average input spike counts over all the training input images for different coding methods in 100 ms time window.

The input spike patterns and average input spike counts over all the training images obtained from different coding methods are shown in Figure 3. The spike patterns were converted from an example digit five. In the rate coding scheme, the spike pattern consists of random spikes in the time window. The spike frequency for each input neuron is proportional to the pixel intensity. The average spike count changes with time with the mean of around four, and the total count in 100 ms time window is 689. In the TTFS coding scheme, each input produces only one spike, and the latest spike appears before 20 ms. The total count is 166. Phase coding scheme generates periodic spike patterns, leading to the highest spike count of 20,325. In the burst coding scheme, each input neuron outputs a burst of spikes or a single spike, depending on the pixel intensity. A large intensity results in a high-frequency burst, while a small intensity produces a sparse burst or a single spike. The latest spikes appear before 20 ms. The total count is 598. Clearly, phase coding generates the most input spikes, while TTFS coding has the fewest input spikes.

### Classification and Computational Performance

The SNN training process is described as follows. An input dataset (MNIST or Fashion-MNIST) is divided into 60,000 training images and 10,000 inference images. Each input image is converted into spikes by different coding schemes within a time window (or latency) ranging from five to 200 ms. The latency is optimized for all the coding schemes. Images are processed sequentially. Learning happens in the synaptic weights between the input layer and excitatory neuron layer. Weights are updated by the STDP rule. 10 epochs of training process are run for each coding scheme. Label assignment and inference is performed after each epoch of training process.

**FIGURE 4:**
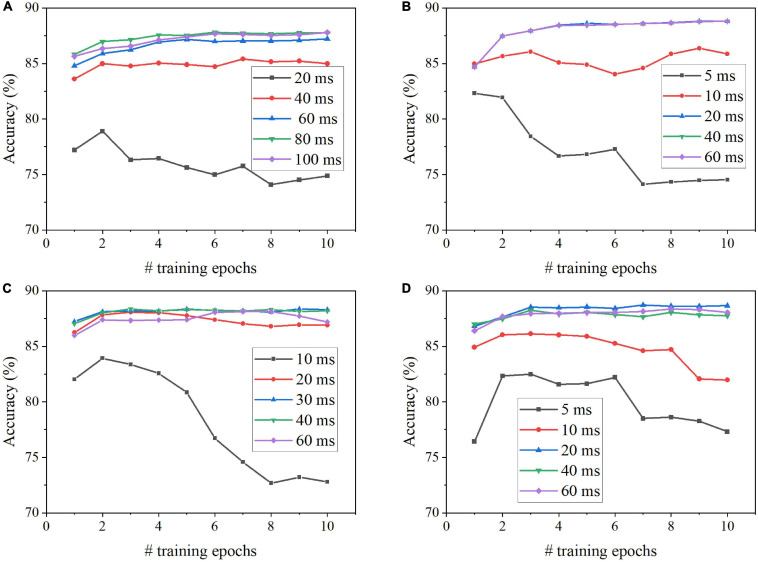
Classification accuracy on MNIST dataset after different numbers of training epochs for different training latencies in the SNN with **(A)** Rate coding, **(B)** TTFS coding, **(C)** Phase coding, and **(D)** Burst coding.

We evaluated the coding schemes on the image classification tasks using MNIST and Fashion-MNIST datasets. The classification performance of each coding scheme was based on classification accuracy and processing latency during training and inference phases. Figure 4 shows the classification results on MNIST dataset for each scheme, including the classification accuracy for different numbers of training epochs and training latencies. The training latency is defined as the time duration of training the SNN with each input image. For rate coding, the accuracy is improved when the training latency is increased up to 80 ms, while 20 ms training latency is long enough for TTFS coding to produce the highest accuracy (Figures 4A,B). Both methods require four epochs of training to reach the highest accuracy. For phase coding, the accuracy increases with the training latency of less than 60 ms and decreases afterward (Figure 4C). This is because, with a longer training latency, the weights associated with the neurons that fire actively at the beginning of the training are updated more frequently, causing these neurons to dominate the competitions. The same behavior can be observed in the case of burst coding, but the drop is less significant after 20 ms training latency (Figure 4D). The optimal training latency is 30 ms for phase coding and 20 ms for burst coding. Both methods require three epochs of training to reach the highest accuracy.

**FIGURE 5:**
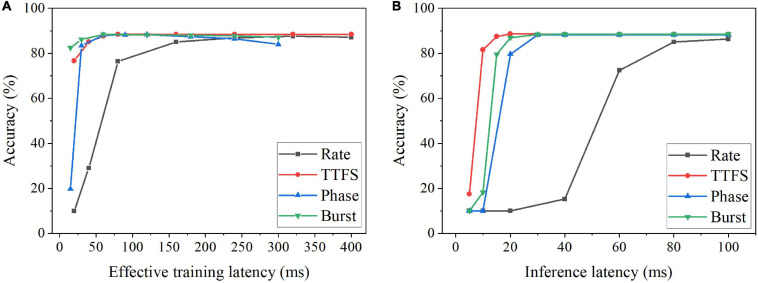
Classification accuracy on MNIST dataset for different coding schemes at different **(A)** effective training latency and **(B)** inference latency. The effective training latency is defined as the training latency multiplied by the number of epochs required to achieve the best accuracy.

Since the optimal training latency and number of epochs vary among these coding schemes, we defined the effective training latency as the product of the training latency and the optimal number of epochs for a fair comparison among the coding schemes. The accuracy change with the effective training latency is shown in Figure 5A for all the coding schemes. Burst coding shows the fastest training convergence speed, while rate coding gives the worst training convergence speed. The inference convergence results are shown in Figure 5B. In this case, TTFS coding leads to the fastest inference convergence speed, while rate coding still largely lags. These results reveal the disadvantage of rate coding, which is the need of a long latency to encode precise information. TTFS coding uses the precise timing of the first spike to represent the information, enabling the fastest information transmission during inference. However, TTFS coding requires a higher training latency than burst coding because training requires a large number of spikes for weight updates. Phase coding also requires a higher latency to encode the information at different phases.

The average simulation runtimes of the SNN with different coding schemes were monitored for processing each image from MNIST dataset during both training and inference phases. The simulations were run in Python language in a single CPU process. The number of spikes and SOPs was also computed. The results are listed in Table 3. The best accuracy achieved by each coding scheme and the required effective training and inference latencies are included for a better comparison. TTFS coding leads to the highest accuracy. It requires the least inference latency and runtime, and the lowest number of spikes and SOPs during both training and inference phases. TTFS coding requires 4x and 7.5x lower processing latency and 3.5x and 6.5x fewer SOPs than rate coding during the training and inference process, respectively. These advantages are attributed to the fact that TTFS coding uses only the first spike and the precise timing for information coding. However, the effective training latency for TTFS coding is longer than those for phase and burst coding. Because of the high training latency, the training simulation runtime for TTFS coding is the same as that for burst coding, even though burst coding uses many more spikes and SOPs. While phase coding has short training and inference latencies, it results in the worst simulation runtime because of the large number of spikes and SOPs. Rate coding gives the lowest accuracy and requires very long latency to reach the convergence. In addition, all the simulations were also run in the SNNs trained and tested on Fashion-MNIST dataset. The results are listed in Table 4. TTFS coding, phase coding, and burst coding show the best accuracy, while rate coding still gives the worst accuracy. The results further confirm the same observations and analyses made in the case of MNIST dataset. Therefore, we can conclude that TTFS coding shows the best overall classification performance, while phase coding and rate coding show the worst classification performance.

**TABLE 3:** Comparison of classification and computational performance on MNIST dataset among different neural coding schemes.

	Rate	TTFS	Phase	Burst
Accuracy (%)	87.46	88.57	88.18	88.39
Effective latency (ms) (Training/inference)	320/150	80/**20**	90/30	**60** /30
Simulation time/image (ms) (Training/inference)	3.79/6.36	**1.10/0.78**	4.29/6.93	1.11/1.01
# spikes (× 10^7^) (Training/inference)	12.594/0.997	**3.565/0.152**	68.150/5.782	10.134/0.570
# SOPs (× 10^8^) (Training/inference)	130.785/9.932	**37.300/1.506**	690.072/57.798	104.947/5.679

*Being bold indicates that this value is the best result in this row.*

**TABLE 4:** Comparison of classification and computational performance on Fashion-MNIST dataset among different neural coding schemes.

	Rate	TTFS	Phase	Burst
Accuracy (%)	68.29	71.31	**71.36**	71.27
Effective latency (ms) (Training/inference)	320/150	80/**20**	**60** /30	**60** /30
Simulation time/image (ms) (Training/inference)	3.95/7.65	**1.28/0.91**	6.89/13.23	1.34/1.55
# spikes (× 10^7^) (Training/inference)	27.471/2.152	**9.393/0.393**	48.502/12.204	23.795/1.326
# SOPs (× 10^8^) (Training/inference)	281.481/21.516	**95.583/3.921**	492.870/121.904	242.611/13.253

*Being bold indicates that this value is the best result in this row.*

For rate coding and burst coding, the classification and computational performance relies on the choice of the model hyperparameters, namely the scaling factor λ and the maximum burst spikes *N*_*max*_, respectively. Therefore, we justify our choice of the model hyperparameters used in the comparisons. The values of the model hyperparameters are selected for the optimal classification and computational performance based on a figure of merit (FOM), which is defined as,

$$F\,O\,M=\frac{\text{Accuracy}}{\mathrm{Total}\,\mathrm{latency}\times \#\,\mathrm{total}\,\mathrm{spikes}\times \#\,\mathrm{total}\,\mathrm{SOPs}}$$

where the total refers to the sum of the values for training and inference, and each quantity is normalized over the corresponding maximum value. By optimally selecting the hyperparameters, we guarantee the fairness of the comparison. Table 5 summarizes the comparison of classification and computation performance on MNIST dataset for different λ. Decreasing λ from eight to four, i.e., increasing the firing rate, can increase the accuracy by around 1%, while further decreasing does not lead to substantial improvement of accuracy. A larger λ requires higher training and inference latency. A smaller λ can only decrease the inference latency but requires more training spikes and operations. Therefore, according to the FOM, the choice of the scaling factor λ as four is the optimal. For burst coding, Table 6 summarizes the classification and computational performance for different *N*_*max*_. Clearly, larger *N*_*max*_ leads to more spikes and SOPs. When *N*_*max*_ is increased from five to 10, no improvement can be observed in the performance. While decreasing *N*_*max*_ from five to two reduces the number of spikes and SOPs, it prolongs both training and inference duration more significantly and also lowers the classification accuracy. Thus, according to the FOM, *N*_*max*_ = 5 is the optimal choice.

**TABLE 5:** Comparison of classification and computational performance on MNIST dataset among different scaling factors λ in rate coding.

Scaling factor λ	2	4	8
Effective latency (ms) (Training/inference)	320 / 80	320 / 150	640 / 200
Accuracy	87.77%	87.46%	86.40%
# spikes (× 10^7^) (Training/inference)	25.101/1.0609	12.594/0.997	12.564/0.6628
# SOPs (× 10^8^) (Training/inference)	260.941 / 10.599	130.785/9.932	131.473/6.618
FOM	1.84	5.81	3.36

**TABLE 6:** Comparison of classification and computational performance on MNIST dataset among different *N*_*max*_ in burst coding.

Maximum number of spikes N_max_	2	5	10
Effective latency (ms) (Training/inference)	400 / 60	60 / 30	60 / 30
Accuracy	88.09%	88.39%	88.20%
# spikes (× 10^7^) (Training/inference)	6.110/0.257	10.134/0.570	15.005 / 1.073
# SOPs (× 10^8^) (Training/inference)	66.112 / 2.562	104.947/5.679	154.611 / 10.721
FOM	5.36	10.14	4.51

### Hardware Implementation of Coding Schemes

**FIGURE 6:**
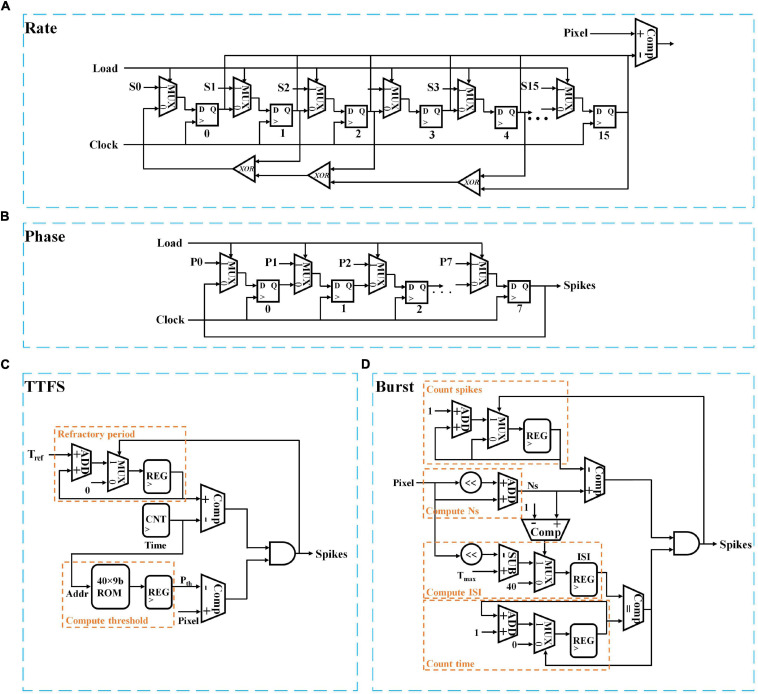
Digital implementations of different neural coding schemes; **(A)** Rate coding, **(B)** Phase coding, **(C)** TTFS coding, and **(D)** Burst coding. S and P stand for a 16-bit seed and an 8-bit input pixel. The clocks in panels **(C,D)** are omitted for simplicity.

In this section, we will discuss the hardware implementation details of each coding scheme in terms of area and power consumption. For the area estimation, the implementation of each coding scheme is considered for general digital systems which are commonly used in state-of-art neuromorphic chips such as Loihi and TrueNorth (Merolla et al., 2014; Davies et al., 2018). The gate-level implementations of all the coding schemes are illustrated in Figure 6. For better comparison, we estimate the equivalent number of NAND gates for each digital gate and use the total number of equivalent NAND gates as a criterion of the hardware area for each coding scheme. The estimation of NAND gate count is based on the designs reported in Weste and David (2015) and summarized in Table 7 for N coding modules.

**TABLE 7:** Hardware implementation results of neural coding schemes.

Gates and power	Rate	TTFS	Phase	Burst
1b-ADD	0	12N	0	21N
1b-SUB	0	0	0	10N
8b-MUL	0	1	0	0
1b-XOR	3N	0	0	0
1b-AND	0	N	0	N
4b-Comp	4N	4N	0	4N
1b-MUX	0	6N	8N	15N
1b-Reg	16N	12N+18	8N	15N
40 × 9b-ROM	0	2	0	0
NAND	316N	340N+1703	76N	544N
Power (mW)	1.14N	0.70N+0.93	2.26N	1.27N

*N is the number of coding modules.*

The rate coding module is implemented with a pseudo-random number generator and a comparator. In Figure 6A, the random number generator was realized in a 16-bit linear feedback shift register with three XOR gates. The total equivalent NAND gate counts are 348 for a single coding module. The implementation of phase coding is the simplest, which only requires multiplexers and 8-bit registers to generate the periodic binary representation of the input, as shown in Figure 6B. The total NAND gate count is 76. TTFS coding can be implemented in two parts. One is used to generate the time-decaying threshold and spike weight, while the other is responsible to generate the first spike. The implementation of exponential functions is complex in a digital hardware system. We adopt a simple and popular implementation method that uses memory tables (ROMs) to store the pre-calculated values. Since the training window of TTFS coding is 20 ms, and the time step is 0.5 ms, the depth of the memory table is set as 40. We use nine bits to represent the threshold and spike weight with one bit for the integer part and eight bit for the fractional part. A multiplier is required to compute the synaptic input, as indicated in Equation (2). Figure 6C only shows the block for computing the threshold. The same design is applied for computing the spike weights. The spike generation is realized by comparing the input and the generated threshold and setting the refractor period of firing neurons as a large number, T_ref_. From the table, the spike generation results in 340 NAND gates. The threshold and spike weight generation unit amounts to 1,703 NAND gates. Since the threshold and spike weight are shared by all the coding modules at each time step, the equivalent gate count is not scaled with N. Thus, when N is large, the overhead added by the threshold and spike weight generation unit can be neglected. Burst coding requires the conversion of the input into an ISI and N_s_ according to Equation (3), and the generation of a spike burst is controlled by the ISI and N_s_. In Figure 6D, the counting spikes block monitors the number of the generated spikes. The counting time block records the number of time steps before generating the next spike. A spike is generated on the condition that the number of the generated spikes is smaller than N_s_ and the interval between two consecutive spikes is ISI. The total equivalent NAND gate count is 544. When *N* ≤ 8, TTFS coding causes the largest area. Whereas, when N is larger, burst coding causes the largest area. Phase coding has the smallest area.

Furthermore, the power consumption in each coding scheme was estimated by implementing each coding scheme on a Xilinx VC709 FPGA board at 100 MHz clock frequency. For TTFS coding, the power consumption by the spike generation unit is 0.7 mW, and the power consumption by the threshold and spike weight generation unit is 0.93 mW for the exponential implementation. Phase coding has the highest power consumption because of the highest spiking activity. When *N* ≤ 2, the power consumption of TTFS coding is higher than rate coding. However, when N is larger, TTFS coding has the lowest power consumption.

### Input Noise Study

**FIGURE 7:**
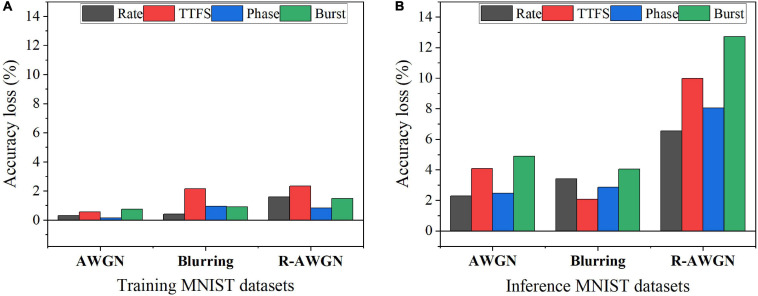
Accuracy loss on MNIST dataset with different types of noise in two noisy scenarios: **(A)** Training with noisy datasets and inference with the no-noise dataset and **(B)** Training with the no-noise dataset and inference with noisy datasets.

To study the impact of input noise, we have applied three types of noisy MNIST datasets in the SNNs with different coding schemes during training and inference. Three types of noise were added to MNIST dataset, such as additive white Gaussian noise (AWGN), motion blurring, and reduced-contrast AWGN (R-AWGN) (Basu et al., 2017). The AWGN has the signal-to-noise ratio of 9.5, emulating background clutter. The motion blur was created with a linear motion of a camera by five pixels with an angle of 15 degrees in the counterclockwise direction. For R-AWGN, the contrast range was scaled down to half and an AWGN with the signal-to-noise ratio of 12 was applied. R-AWGN imitates the background clutter with significant change in lighting conditions. The results of the accuracy loss are shown in Figure 7. The accuracy loss was computed with reference to the accuracy on the no-noise dataset for the SNN trained with the no-noise dataset after 10 epochs of training. For each coding scheme, the optimal training latency and inference latency were used to process each input image. Two scenarios of the presence of input noise were analyzed. The first scenario was to introduce the input noise during the training phase and perform inference with the no-noise MNIST dataset. The corresponding results are shown in Figure 7A. The motion blur noise and R-AWGN have the worst effect on the performance of the neural coding schemes. TTFS coding is most sensitive to the motion blur and R-AWGN, while phase coding has the best resilience to all the types of input training noise. In the second scenario, the training was performed with the no-noise MNIST dataset, and the input noise was introduced during the inference phase. The corresponding results are shown in Figure 7B. The overall accuracy loss for all the coding schemes becomes much higher than that in the first scenario, suggesting that the training could help mitigate the negative impact of the input noise. However, for TTFS coding, the accuracy loss caused the blurring remains the same in both scenarios, suggesting that TTFS coding is most sensitive to blurring noise. The accuracy loss resulting from burst coding is the most severe for all the noisy datasets. Rate coding and phase coding have the best resilience to various types of input inference noise. In summary, phase coding shows the highest resilience to various input noise types during both training and inference, while TTFS coding and burst coding show the worst resilience.

**FIGURE 8:**
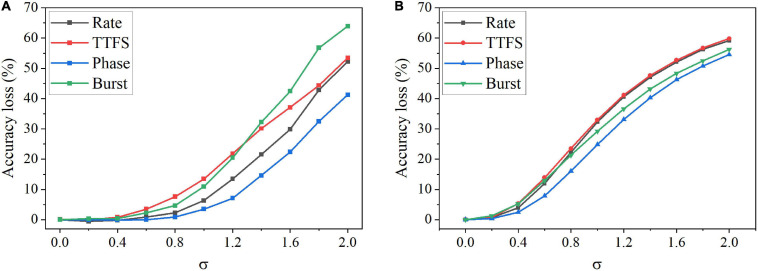
Accuracy loss on MNIST dataset after adding the AWGN with different standard deviations, σ. **(A)** The noise was added to the training images, and **(B)** the noise was added to the inference images.

Furthermore, we studied the impact of the input noise variation on different coding schemes. The AWGN with different standard deviations was added to the training and inference dataset images separately. Figure 8A shows the accuracy loss after the noise was added to the training images. The noise has the largest impact on burst coding and TTFS coding schemes as the deviation increases. Phase coding shows the highest resilience. In Figure 8B, when the noise was introduced during inference, the impact was reduced on all the coding schemes. Phase coding still shows the highest resilience. TTFS coding and rate coding are the worst affected. In phase coding scheme, the noise effect was reduced by the spike weight that decreases with the increasing phase since most of the noise values are small, and the resulting noisy spikes appear in the large phases. In TTFS coding scheme, the input information is carried by the times of the first spikes, and the noise can easily disturb the timings. Small noise values can cause large errors in the representation. Therefore, phase coding is the most resilient scheme to both training and inference input noise, while TTFS coding has the worst overall resilience.

### Weight Pruning

Computations in neural networks involve a massive number of parameters that drastically scale up with the number of neurons and layers, which imposes a huge burden on hardware resources, limits the processing speed, and consumes a large amount of energy. Network compression techniques were proposed to tackle these challenges. Pruning and quantization are the most favorable and efficient network compression techniques because of their simple implementation and high effectiveness. In this section, we study the impact of weight pruning on the coding schemes and evaluate the capability of each coding scheme to achieve efficient network compression.

**FIGURE 9:**
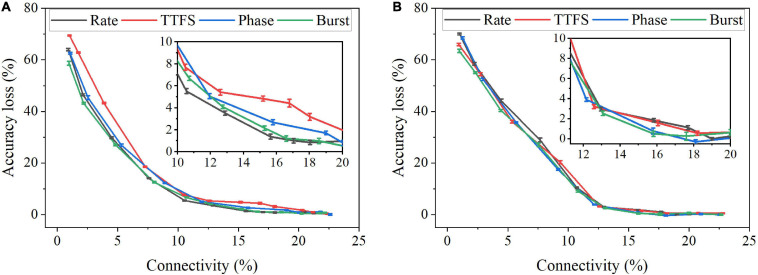
Accuracy loss on MNIST dataset changes with network connectivity resulting from weight pruning. **(A)** An online weight pruning method and **(B)** a post-training weight pruning method were considered.

In this work, we considered two weight pruning methods; an online pruning method and a post-training pruning method. In the online pruning method, a constant weight pruning threshold was applied while the training was in process. The pruning process started after a short training phase that involved 30,000 training images and continued for 10 training epochs. The pre-pruning training phase was enforced to ensure that the network learned major input features (Guo et al., 2020a). In the post-training pruning method, the SNN was trained for 10 epochs, and the pruning was performed before inference. Various thresholds were used. Both network connectivity and accuracy decreased with the increasing pruning threshold. The connectivity was defined as the percentage of the unpruned weights over the total weights. Figure 9A displays the pruning results for the online pruning method. 10 simulations were run with different random seeds. Error bars are plotted as the standard errors of the data mean. Around 85% of weights can be pruned without causing significant accuracy loss. From the inset plot, we can see that the largest difference exists between TTFS coding and rate coding, ranging from 2 to 4% when the connectivity is larger than 10%. As the connectivity drops below 10%, the largest difference is between TTFS coding and burst coding, which can go up to 10%. The difference between TTFS coding and other schemes during training is distinguishable. The difference among the other three coding schemes is mostly less than 1% and thus can be negligible. Figure 9B shows the pruning results for the post-training pruning method. The difference between these coding schemes is negligible. Therefore, TTFS coding is least capable of achieving efficient network compression by conducting pruning while training.

### Weight Quantization

**FIGURE 10:**
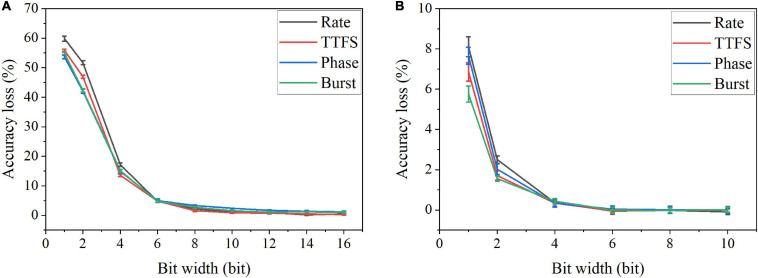
Accuracy loss on MNIST dataset in the SNN with different coding schemes after weight quantization **(A)** during training and **(B)** post training.

Weight quantization brings in a huge benefit in the reduction of memory size and energy consumption. However, quantization induces numerical errors and limits the precision of arithmetic computation. For the implementation of weight quantization, we used stochastic rounding (SR) method because this method ensures a non-zero probability that a small weight update will not be rounded to 0 (Gupta et al., 2015). The SR method rounds the number x to the fixed-point number with a probability proportional to the difference between them, which is described by

$$S\,R\,\left(x\right)=\left\{ \begin{array}{c} \left\lfloor x\right\rfloor ,w.p\,r\,o\,b\,. 1-\frac{x-\left\lfloor x\right\rfloor }{\epsilon },\\ \left\lfloor x\right\rfloor +\epsilon ,w.p\,r\,o\,b.\frac{x-\left\lfloor x\right\rfloor }{\epsilon } \end{array}\right.$$

where ϵ is the precision of the fixed-point representation, and ⌈*x*⌉ is the largest integer multiple of ϵ less than or equal to x. We applied SR method for weight quantization in the SNN during training and post-training. The simulation results of accuracy loss caused by quantization for different coding schemes are shown in Figure 10. The results for the training phase were obtained after 10 epochs. Error bars were added after 10 simulations for each scheme. From Figure 10A, severe accuracy drop happens when the bit width is reduced below six bits for quantization during training. For post-training quantization, significant accuracy loss is observed when the bit width is less than two bits (Figure 10B). Rate coding can be seen to have the worst accuracy loss in both cases, while burst coding has the smallest loss when the bit is smaller than four bit.

### Impact of Synaptic Noise and Fault

Although digital implementations are widely used for neuromorphic systems, their performance is limited in the current machine learning applications. Analog computation paves the way to achieve Tera operations per second per watt efficiency which is 100x compared to digital implementations (Gil and Green, 2020). Various types of analog devices have been used to implement neural networks, such as CMOS transistors (Indiveri et al., 2011), floating gate transistors (Kim et al., 2018a), gated Schottky diode (Kwon et al., 2020), and emerging memory devices (like PCM, RRAM, STTRAM) (Kim et al., 2018b). Despite the potential of these devices in analog computation, they suffer from many non-idealities which could limit the performance, such as limited precision, programming variability, stuck-at-fault (SAF) defects, retention and others (Fouda et al., 2019). In this work, we study the impact of two main types of synaptic variations, namely, synaptic noise and synaptic SAF defects, which are induced by device non-idealities existing in analog hardware.

Synaptic noise is mainly induced when programming synaptic analog devices (Sheng et al., 2019). To test the noise resilience of the coding schemes, we assume that all the synaptic weights are stored in synaptic devices. The synaptic noise mostly results from programming noise when weight writes are performed. During training, we added Gaussian noise to each quantized weight update to model the effect of programming noise on the weights stored in synaptic devices. The model is described by

$$w+=Q\,\left(\text{Δ}\,w\right)+N\,\left(0,{\left(\sigma \,\epsilon \right)}^{2}\right)$$

where *w* are the weight, Δ *w* is the weight change obtained from the STDP function, *Q*(⋅) is the quantization function, ϵ is the precision of the fixed-point representation, and σ is the percentage.

**FIGURE 11:**
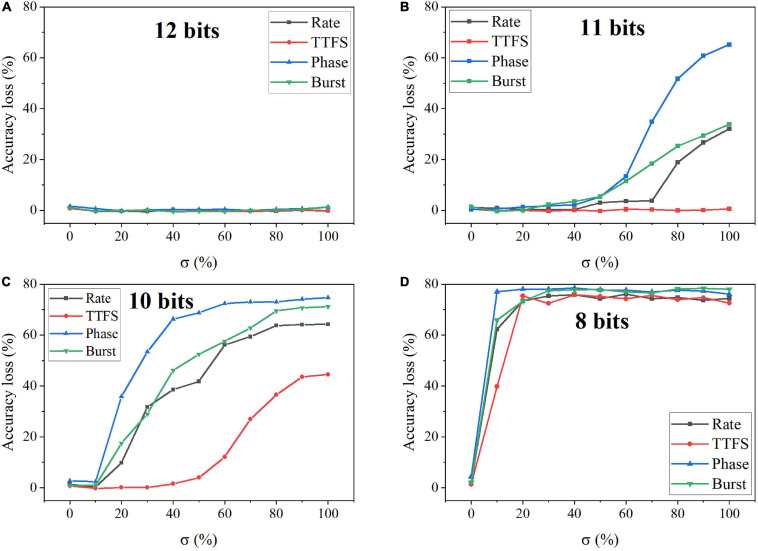
Accuracy loss on MNIST dataset in the SNN with different coding schemes after adding programming noise to the quantized weight updates during training. The quantized bit width was changed from **(A)** 12 bits, **(B)** 11 bits, **(C)** 10 bits, to **(D)** eight bits.

**FIGURE 12:**
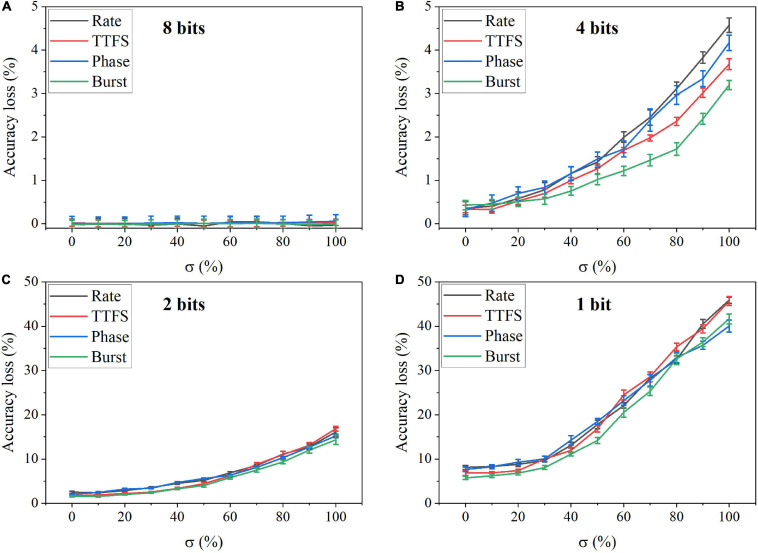
Accuracy loss on MNIST dataset in the SNN with different coding schemes after adding programming noise to the quantized weights post-training for **(A)** eight bits, **(B)** four bits, **(C)** two bits, and **(D)** one bit.

In the simulation, we changed the value of σ from 0 to 100% for four bit widths. The accuracy loss results on the MNIST dataset were obtained after 10 training epochs and are shown in Figure 11 for different coding schemes. In the case of 12 bits, the noise has no adverse effect on the accuracy (Figure 11A). Due to small precision, the added noise becomes small. When the bit width is decreased from 12 bits to eight bits, the accuracy loss starts to grow with the noise level, as shown in Figures 11B–D. Particularly, with 8-bit width, the network fails to learn input features even when a small noise was added. Phase coding causes the most severe accuracy loss, while TTFS coding has the best resilience. This phenomenon can be explained by the number of weight updates resulting from the coding schemes. Because of the highest spike activity, phase coding causes the most updates during training. Whereas, TTFS coding has the least updates. Since the noise comes with each update, TTFS coding is least affected. There is a small overall performance difference between burst coding and rate coding because they require a similar number of updates. Moreover, the impact of post-training programming noise was considered. In this case, the SNN was trained offline without any synaptic noise. The well-trained weights were quantized with the SR method and mapped onto synaptic devices. During the mapping, the programming noise was added to the quantized weights. Similarly, we changed the value of σ in the noise model from 0 to 100% for different quantization bit widths. The results of the accuracy loss on MNIST dataset are shown in Figure 12 for different coding schemes. Error bars were added after 10 simulations for each scheme. Obviously, the impact of the post-training synaptic noise is much smaller than that of the training synaptic noise. No loss can be observed in the case of 8-bit quantization regardless of the σ values in Figure 12A. When the bit width decreases from eight bits to one bit, the loss increases with the noise variation, as shown in Figures 12B–D. The error bars are helpful to distinguish the burst coding from the other coding schemes. We can claim that burst coding shows the best resilience to the added post-training synaptic noise. The difference among other coding schemes is negligible. The largest loss difference increases from 1.4 to 4.0%, with the bit width decreasing from four bits to one bit.

**FIGURE 13:**
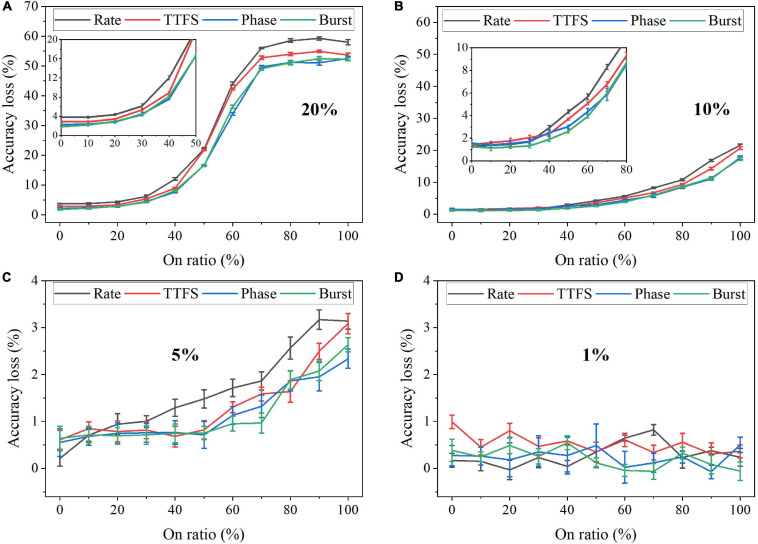
Accuracy loss on MNIST dataset in the SNN with different coding schemes after considering the stuck-at-fault model during training with the fault rate of **(A)** 20%, **(B)** 10%, **(C)** 5%, and **(D)** 1%.

**FIGURE 14:**
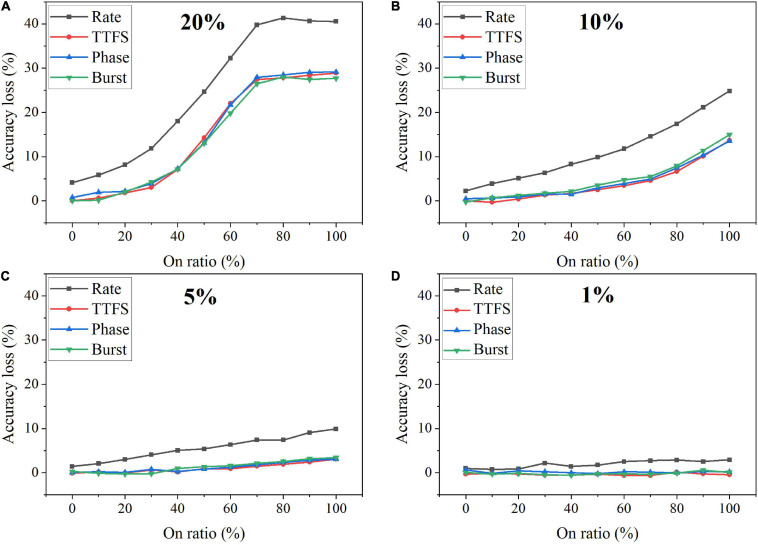
Accuracy loss on Fashion-MNIST dataset in the SNN with different coding schemes after considering the stuck-at-fault (SAF) model during training with the rate of **(A)** 20%, **(B)** 10%, **(C)** 5%, and **(D)** 1%.

Faulty devices are generally encountered in analog computing systems, due to many reasons, such as fabrication process variations, spot defects, aging phenomenon, mechanical stress, heavy device testing and utilization, etc. (Lewyn et al., 2009; Vatajelu E.I. et al., 2019; El-Sayed et al., 2020). Vatajelu E. et al. (2019) reported and analyzed different generic fault models that could exist in SNN hardware implementation. We chose the synaptic SAF model in this study since it appears very often in hardware, especially in the promising and newly emerging analog devices, and it has a profound impact on hardware performance (El-Sayed et al., 2020; Kwon et al., 2020; Zhang B. et al., 2020). A SAF device has its conductance state fixed at either a high or low conductance state. We applied the SAF defect model in our simulation to demonstrate the degree of fault tolerance the coding schemes can bear. Four different fault rates were used during training, namely, 20%, 10%, 5%, and 1%. We changed the ratio of the stuck-on devices (on ratio) over all the fault devices from 0 to 100% at each fault rate. The resulting accuracy loss on MNIST dataset was obtained after 10 training epochs and is shown in Figure 13. Error bars were added after 10 simulations for each scheme. When the fault rate is larger than 1%, the accuracy loss increases with the on ratio, suggesting that the network prefers stuck-off fault devices. This is mainly because the input patterns are very sparse, filled mostly with 0 s. The error bars are useful to distinguish rate coding from the other coding schemes in the case of 5% fault rate. Rate coding has the worst synaptic fault tolerance during training. When the fault rate is larger than 5%, it can be clearly seen that rate coding has the largest loss. The difference among these coding schemes is diminished when the fault rate decreases to 1%. For example, the difference between rate coding and burst coding at 50% on ratio decreases from 6 to 1%, with the fault rate decreasing from 20 to 5%. With 1% fault rate, there is no difference since these 1% weights have a negligible impact on the network performance. We have also investigated the impact of SAF defect on the coding schemes during inference. There is less than 1% difference in the accuracy loss among them. Moreover, the network was also tested with Fashion-MNIST dataset. Since the input patterns in the Fashion dataset are more complex than those in MNIST dataset, the impact of synaptic faults on these coding schemes becomes more obvious, and hence the difference among these coding schemes becomes more significant, as shown in Figure 14. The largest difference among the coding schemes at 50% on ratio decreases from 11 to 2%, with the fault rate decreasing from 20 to 1%. These results further confirm that rate coding is most susceptible to the synaptic fault. This could be due to that the SAF defect could happen to any device and hence the SAF fault devices were randomly selected before training starts, which induces randomness in the weights and hence adds more uncertainty in rate coding that encodes information in stochastic spike trains. Therefore, we can conclude that rate coding has the worst synaptic fault tolerance during training, while the other coding schemes have similar performance.

## Comparison and Discussion

**FIGURE 15:**
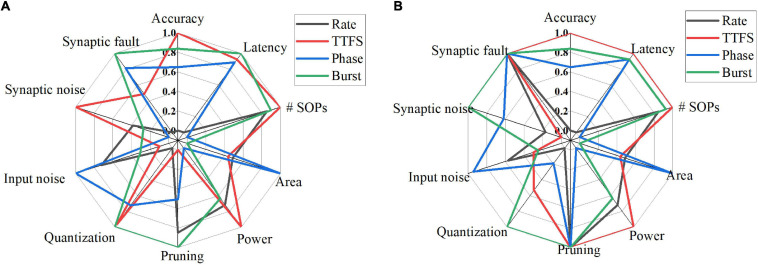
Quantitative comparisons among different coding schemes from various aspects for **(A)** training and **(B)** inference. In each dimension, the data were normalized with the min-max normalization method. In the cases of pruning, quantization, input noise, synaptic noise, and synaptic fault, the average accuracy loss for each coding scheme was used. The greater value, the better.

To provide a comprehensive comparison among different coding schemes, we summarized their performance in 10 aspects for both training and inference phases, as shown in Figure 15A,B. In each dimension, the data point for each coding scheme was obtained by normalizing the accuracy loss with the min-max normalization method, which is given by (*x*_*max*_−*x*_*i*_)/(*x*_*max*_−*x*_*min*_), where *x*_*i*_ is the accuracy loss of the *i*-th coding method, *x*_*max*_ is the maximum value among the coding methods, and *x*_*min*_ is the minimum value. In Figure 15A, the latency refers to the effective training latency. In the cases of pruning, quantization, input noise, synaptic noise, and synaptic fault, the average accuracy loss for each coding scheme was normalized and plotted for comparison. A greater value in each dimension leads to better performance. For pruning and quantization, the average accuracy loss across the whole range was computed and normalized for each coding scheme. The average accuracy loss at the 10-bit width was computed and normalized for synaptic noise during training. The results for eight bits and 11 bits can also be used to compute the loss since they showed the same performance order among these methods. The average accuracy loss at the 1-bit width was used for synaptic noise during inference. In the case of input noise, both noise type and noise variations were considered. To evaluate the overall resilience to different noise types, we used the average accuracy loss on all the noisy datasets for each coding scheme. For noise variations, the average loss was computed. Then, the normalized loss for each coding scheme was obtained by taking the average over the two normalized loss values. For synaptic fault, the results of a 20% fault rate were used, and the average loss was computed.

Tables 8, 9 summarize the qualitative comparisons among different coding schemes according to the results in Figure 15. The more the number of check marks is, the better performance the coding scheme has in each category. During the training phase, rate coding has good compression performance by pruning, good resilience to input noise. But it suffers from the lowest accuracy, the highest latency, the worst compression performance by quantization, and the worst fault tolerance. TTFS coding has the smallest number of SOPs and power consumption, least susceptible to the synaptic noise. But it has large area, the worst compression effectiveness by pruning, lowest resilience to input noise, and bad tolerance to synaptic fault. Phase coding shows the smallest area, good compression performance by quantization, the highest input noise resilience, good synaptic fault tolerance. But it has the largest number of SOPs and power consumption, and the worst synaptic noise resilience. Burst coding shows the shortest latency, small number of SOPs and power consumption, the best compression performance by pruning and quantization, and the best synaptic fault tolerance. But it has the disadvantage of the largest area and poor noise resilience.

**TABLE 8:** Qualitative comparisons among different coding schemes from various aspects for training.

	Rate coding	TTFS coding	Phase coding	Burst coding
Accuracy	√	√√√√	√√	√√√
Latency	√	√√√	√√	√√√√
# SOPs	√√	√√√√	√	√√√
Area	√√√	√√	√√√√	√
Power	√√√	√√√√	√	√√
Pruning	√√√	√	√√	√√√√
Quantization	√	√√√	√√	√√√
Input noise	√√√	√	√√√√	√√
Synaptic noise	√√√	√√√√	√	√√
Synaptic fault	√	√√	√√√	√√√√

**TABLE 9:** Qualitative comparisons among different coding schemes from various aspects for inference.

	Rate coding	TTFS coding	Phase coding	Burst coding
Accuracy	√	√√√√	√√	√√√
Latency	√	√√√√	√√√	√√√
# SOPs	√√	√√√√	√	√√√
Area	√√√	√√	√√√√	√
Power	√√√	√√√√	√	√√
Pruning	√	√	√	√
Quantization	√	√√√	√√	√√√√
Input noise	√√√	√√	√√√√	√
Synaptic noise	√√	√	√√√	√√√√
Synaptic fault	√	√	√	√

During the inference phase, the difference among these schemes in all the dimensions on the left half circle becomes less significant. In the cases of pruning and synaptic fault, there is no difference among these schemes. Area and power consumption are the same as in the training phase. Rate coding has good resilience to input noise but suffers from the highest latency and the worst compression performance by quantization. TTFS still holds the same advantages except for the worst synaptic noise resilience. The resilience of phase coding to synaptic noise becomes better than rate coding and TTFS coding. For burst coding, the compression performance by quantization becomes the best.

Clearly, based on the discussion above, no coding scheme is perfect in all aspects, and each coding scheme has its advantages and drawbacks. The choice of the neural coding scheme depends on the constraints and considerations in the design. This comparative analysis of different neural coding schemes shows how to select the best coding scheme in different scenarios. For example, if computational performance and hardware performance are the primary concern in the design, the best choice would be TTFS coding. If network performance is largely affected by input noise, the best choice would be phase coding. If network compression is the main consideration, the best choice would be burst coding. If the network performance is largely limited by hardware non-idealities, the best choice would be burst coding. It is worth mentioning that due to the simplicity of the rate coding, SNNs and neuromorphic hardware have mainly relied on rate coding without investigating other coding techniques. Our study shows that the other coding schemes can outperform rate coding in many aspects which prove that rate coding is not always the best choice.

In the previous work, different neural coding schemes were compared in terms of classification accuracy, latency, the number of spikes, and energy during inference (Park et al., 2020). The comparison revealed that TTFS coding won against the other coding schemes in classification and computational performance. The advantage of TTFS coding was expected because it used precise timing and only one spike. Our work also demonstrated the excellent performance of TTFS coding during inference in terms of classification performance, computational performance, and power consumption. Most importantly, we looked into the real-time applications of the neural coding schemes in neuromorphic systems and investigated their performance in various aspects, including the hardware implementation, effectiveness of network compression, noise resilience, and fault tolerance. We provided a thorough comparison among these coding schemes and clear guidelines for utilizing different neural coding schemes in neuromorphic systems.

While the performance of the SNN is limited by the shallow structure and unsupervised learning algorithm, the accuracy level can be improved up to 95% by increasing the network size (Diehl and Cook, 2015). The state-of-the-art classification performance in SNNs is achieved through complicated supervised backpropagation (BP) algorithms, such as temporal coding-based methods (Mostafa, 2018; Comsa et al., 2020; Kheradpisheh and Masquelier, 2020; Zhang M. et al., 2020), rate coding-based methods (Lee et al., 2016; Fang et al., 2020) and a generic approach (Neftci et al., 2019). Although SNNs trained with BP algorithms perform closely to ANNs in various recognition tasks, this comes at the cost of long training time, more computation resource, and high computational power, making BP algorithms not suitable for developing neuromorphic systems. Moreover, BP algorithms are not biologically plausible. It is more meaningful in neuroscience to study the impact of neural coding methods with a biological plausible learning algorithms, e.g., STDP algorithm. On the other hand, the high accuracy achieved by STDP in the literatures is due to multiple convolution-pooling layers and a pre-trained supervised classifier (by SVM or reinforcement learning) (Kheradpisheh et al., 2018; Mozafari et al., 2018). However, it is also important to investigate the impact of neural coding methods on deep networks trained with STDP or supervised learning algorithms, as it would be helpful to develop a high-performance computational system. We would like to consider the study in our future work.

We have chosen this network structure and learning algorithm based on the following reasons.

• In the literature, among unsupervised two-layer SNNs, this network structure shows the best performance with an STDP learning rule only (Shi et al., 2019; Tavanaei et al., 2019).
• This network has been widely adopted to study the performance of a biological SNN for different purposes, such as studying different STDP models (Diehl and Cook, 2015), improving STDP learning (Panda et al., 2018), and pruning (Shi et al., 2019; Guo et al., 2020b).
• The network structure uses a combination of different biological plausible models, including the LIF neuron model, conductance-based synaptic model, STDP model, WTA network model, and intrinsic plasticity model. Although the network can only produce limited classification accuracy, it closely mimics biological processes and provides a good platform to study how a biological neural network performs computations unsupervised.
• STDP relies on local information, making it easy to be implemented in hardware and enabling scalable online learning.

Most importantly, this study provides important understanding of the impact of different coding schemes on various aspects of the performance of a neuromorphic system. With this understanding, it is beneficial for neuromorphic system researchers to consider and select corresponding coding schemes to achieve specific design goals. Moreover, we studied the coding schemes in biological SNNs with various plausible neural models. Our results can provide insights for neuroscientists of the interactions between spike coding schemes, neural models and learning capability.

## Conclusion

In this work, we investigated different neural coding schemes in an unsupervised SNN from various aspects during training and inference phases, including classification performance, computational performance, hardware implementation, network compression efficacy, noise resilience, and fault tolerance. These coding schemes were tested on two classification tasks. The classification and computational performance were analyzed in terms of accuracy, latency, and SOPs. To evaluate the impact of these coding schemes on hardware implementation, we have analyzed the implementation for the area and power consumption and the network compression efficacy by pruning and quantization. Considering the presence of input noise in real-time applications, we evaluated the sensitivity of each coding scheme to different types of input noise and the noise variations. Furthermore, synaptic noise and fault exist in analog systems because of device non-idealities. The robustness of each coding scheme to the non-idealities in the circuits was studied and compared. With a thorough analysis of all the aspects, a comprehensive comparison among these schemes was provided, revealing the advantages and disadvantages of each coding scheme. This study has laid out clear guidelines for selecting a neural coding scheme for achieving the best performance in neuromorphic systems.

## Data Availability Statement

The original contributions presented in the study are included in the article/supplementary material, further inquiries can be directed to the corresponding author/s.

## Author Contributions

WG, MF, AE, and KS: conceptualization. WG, MF, AE, and KS: methodology. WG: software, algorithms, and writing-original draft preparation. WG and MF: investigation and validation. MF, AE, and KS: writing-review and editing. AE and KS: supervision. All authors contributed to the article and approved the submitted version.

## Conflict of Interest

The authors declare that the research was conducted in the absence of any commercial or financial relationships that could be construed as a potential conflict of interest.
